# Strategies for successful implementation of preliminary image evaluation

**DOI:** 10.1002/jmrs.354

**Published:** 2019-09-15

**Authors:** Gary Denham

**Affiliations:** ^1^ Radiology Department Manning Hospital Taree New South Wales Australia

Radiographers have been providing their opinion on radiographic findings to referring physicians in Australia in an informal capacity for some time. Evidence has shown improved outcomes for patients when radiographers and referring clinicians work collaboratively as part of a multidisciplinary healthcare industry.^1^ Historical hierarchical structures within the health industry have often necessitated the informal methods radiographers employ to communicate radiographic findings to referrers.^2^ Fortunately, the situation is changing with the Queensland Government promoting the benefits of implementing such a system with the release of the ‘radiographer written comment implementation toolkit’.^3^ The toolkit was designed to assist with the implementation of a radiographer commenting system within Queensland Health facilities. The toolkit details strategies for successful implementation and ongoing clinical governance.

A number of barriers have been identified to the implementation of formalised radiographer abnormality detection systems in Australia which include the following: lack of education, lack of confidence and lack of support from radiologist colleagues.^4^ The Australian Society of Medical Imaging and Radiation Therapy (ASMIRT) formed a steering committee to overcome the barriers to implementing formalised radiographer commenting in Australia. The rationale of the Steering Committee was always to improve patient safety through better outcomes. The creation of a credentialing system, though not compulsory, to provide a written comment was one way to overcome some of the barriers identified by Neep et al.^4^ The Steering Committee chose to refer to radiographer commenting as a preliminary image evaluation (PIE) so as to not infer the provision of a radiology report.

In recent times, Logan Hospital in Queensland has set the standard for radiographers to fulfil full scope of practice with the implementation of a formalised PIE system. The prospective longitudinal study by Brown et al.^5^ in this journal is an outstanding example of high‐quality research being conducted within the radiography profession to provide evidence of the benefits of a formalised PIE system within Australia. It is with this evidence that the ASMIRT PIE Steering Committee will be able to develop a robust PIE credentialing system within Australia that can withstand external scrutiny.
